# The African swine fever virus B125R protein antagonizes JAK-STAT signalling by promoting the degradation of IFNAR2

**DOI:** 10.1186/s13567-025-01523-x

**Published:** 2025-04-23

**Authors:** Jun-Hao Fan, Yan-Yan Zhao, Yu-He Ma, Xiao-Ya Pan, Han-Cheng Shao, Meng-Hui Zi, Haojie Ren, Yuhang Zhang, Shichong Han, Bo Wan, Gai-Ping Zhang, Wen-Rui He

**Affiliations:** 1 International Joint Research Centre of National Animal Immunology, College of Veterinary Medicine, Henan Agricultural University, Zhengzhou, 450000 Henan China; 2 Longhu Laboratory, Zhengzhou, 450000 Henan China; 3 Ministry of Education Key Laboratory for Animal Pathogens and Biosafety, Henan Agricultural University, Zhengzhou, 450000 China

**Keywords:** African swine fever virus, JAK-STAT signalling pathway, IFN-β, B125R protein, IFNAR2, autophagy

## Abstract

African swine fever (ASF) is a highly contagious and severe hemorrhagic disease caused by African swine fever virus (ASFV). Currently, few safe and effective vaccines or antiviral drugs are available for its prevention. Interferon (IFN), a key component of innate antiviral immunity, induces interferon-stimulated genes (ISGs) by activating the JAK-STAT signalling pathway, resulting in antiviral effects. ASFV strains, including ASFV SY18, ASFV HLJ18, and ASFV BA71V, are highly sensitive to IFN-I treatment; however, the mechanisms by which ASFV antagonizes the host type I IFN response have not been fully elucidated. In this study, we identified the ASFV B125R protein (pB125R) as a negative regulator of the JAK-STAT pathway. We observed that ectopically expressed pB125R significantly suppressed the IFN-β-triggered activation of JAK-STAT signalling in HEK293T and PK-15 cells. Mechanistic studies revealed that pB125R binds to IFNAR2 and promotes its autophagic degradation, impairing the signal transduction of the IFN response at an early stage. This ultimately reduces the nuclear translocation of the ISGF3 complex and decreases ISG production. Our findings highlight the immunosuppressive activity of pB125R and reveal a novel mechanism by which ASFV evades the host IFN response, contributing to potential strategies for developing vaccines and therapeutics against ASF.

## Introduction

African swine fever (ASF), caused by African swine fever virus (ASFV), is characterized by high fever and severe haemorrhage in multiple organs [1]. First reported in Kenya in 1921 [2], ASF has spread across Africa, Europe, Asia, and the Americas following multiple transcontinental transmissions [2–4]. This disease poses a serious threat to the global pig industry and ecological security, yet effective drugs or vaccines remain elusive. Consequently, strategies for the prevention and control of ASF are critically needed.

ASFV, a member of the *Asfarviridae* family, is the only insect-borne DNA virus [5]. Its complex multilayered structure houses a double-stranded DNA (dsDNA) genome approximately 170–194 kbp long, encoding approximately 150 open reading frames and more than 160 viral proteins [6]. This structural complexity facilitates effective infection and immune evasion mechanisms [7]. However, significant gaps remain in our understanding of the viral proteins that mediate immune evasion and the molecular mechanisms underlying viral pathogenesis, hindering vaccine development.

Type I interferon (IFN-I) is central to the innate antiviral response, promoting the production of interferon-stimulated genes (ISGs) through the activation of the JAK-STAT signalling pathway, thereby impeding viral replication [8]. During viral infections, conserved pathogen-associated molecular patterns (PAMPs) are detected by cytosolic pattern recognition receptors (PRRs), leading to the activation of the transcription factors NF-κB and IRF3, along with the induction of IFNs and proinflammatory factors [9]. Secreted IFN-I binds to the plasma membrane-localized IFN receptor (IFNAR1/IFNAR2), recruiting Janus kinase (JAK) and tyrosine kinase 2 (TYK2). Activated JAKs then phosphorylate signal transducers and activators of transcription (STAT) 1 and STAT2 [10], forming the IFN-stimulated gene factor (ISGF) 3 complex with IFN regulatory factor 9 (IRF9). This complex translocates to the nucleus to initiate *ISG* transcription, where it exerts antiviral effects [11]. ISGs target viral infection and replication processes, effectively neutralizing invading viruses [12].

The virulent ASFV strain Armenia/07 inhibits IFN-I synthesis by negatively regulating the cGAS-STING pathway [13]. Several ASFV-encoded proteins have been reported to be involved in controlling the signalling of the cGAS-STING pathway. ASFV pB318L interacts with the transmembrane region of STING and inhibits its translocation from the ER to the Golgi [14], and ASFV pA151R prevents K63-linked polyubiquitination and TBK1 phosphorylation by degrading the E3 ligase TRAF6 [15]. ASFV pDP96R interacts with a crucial karyopherin (KPNA)-binding site within IRF3, disrupting the KPNA-IRF3 interaction and consequently impeding the translocation of IRF3 to the nucleus [16]. ASFV pMGF505-2R of ASFV inhibits TBK1 phosphorylation and IFN-β production by targeting STING [17]. In addition, both virulent Arm/07/CBM/c2 and the attenuated ASFV strain NH/P68 have been shown to impair the JAK/STAT signalling pathway [18]. Studies have shown that several ASFV proteins are able to target components of the JAK-STAT pathway and thereby inhibit the expression of ISGs. For example, pMGF360-9 L promotes the degradation of STAT1 and STAT2 via the apoptotic and ubiquitin‒proteasome pathways, respectively, thereby suppressing downstream signal transduction [19]. In addition, pMGF505-7R inhibits ISGF3 heterotrimer formation by interacting with IRF9 and prevents the nuclear translocation of ISGF3, leading to the inhibition of the JAK-STAT signalling pathway [20]. The ASFV H240R protein disrupts the interaction between IFNAR1 and TYK2 and between IFNAR2 and JAK1, thereby suppressing ISG production [21].

Taken together, these findings suggest that ASFV infection triggers the production of IFN-I mainly by activating the cGAS‒STING signalling pathway. ASFV strains, including ASFV SY18, ASFV HLJ18 and ASFV BA71V, are highly sensitive to IFN-I treatment [22, 23]. In addition, ASFV achieves immune escape by preventing IFN production as well as the IFN-triggered antiviral response. Viral genes that counteract host innate immunity to ASFV are often associated with virulence. Deletion or mutation of these genes usually results in attenuation of ASFV. For example, ASFV strains lacking MGF360-9 L, MGF505-7R, or H240R exhibit reduced replication capacity and are less pathogenic to pigs than wild-type strains are [19, 24–26], and combined deletion of H240R and MGF505-7R attenuates the virulence of highly virulent ASFV HLJ/18 and provides a greater level of protection against homologous challenges [27]. However, live attenuated virus (LAV) vaccines currently produced by deleting the reported virulence genes of ASFV still cannot fully attenuate this virulence and provide effective protection [28]. Therefore, further investigations to identify viral proteins involved in regulating the JAK-STAT signalling pathway and elucidate the mechanisms by which ASFV modulates IFN responses are urgently needed.

The ASFV B125R protein (pB125R) is a late-transcribed viral protein comprising 125 amino acids, and its biological function remains largely unknown [29]. Our findings reveal that pB125R acts as a negative regulator of the JAK-STAT signalling pathway, significantly inhibiting the IFN-β-induced transcription of antiviral genes and the activation of the STAT1/2 promoter. Mechanistic studies revealed that pB125R promotes the autophagic degradation of IFNAR2, leading to decreased nuclear translocation of ISGF3 and reduced ISG production. These results highlight the immunosuppressive activity of pB125R and elucidate a novel mechanism by which ASFV evades the IFN response, contributing to vaccine and therapeutic development against ASF.

## Materials and methods

### Cells

HEK293T cells were kindly provided by Prof. Hong-Bing Shu, while PK-15 cells were obtained from the American Type Culture Collection. Both HEK293T and PK-15 cells were cultured in Dulbecco’s modified Eagle’s medium (DMEM; Solarbio, Beijing, China) supplemented with 10% foetal bovine serum (FBS; Nulen, Shanghai, China) at 37 °C in a 5% CO_2_ incubator. To create stable cell lines overexpressing the B125R protein (pB125R), we employed lentivirus-mediated gene editing. Briefly, HEK293T cells were transfected with the packaging plasmids psPAX2 and pMD2.0G and either the donor plasmid pLOV-B125R or the empty vector pLOV. After 36–48 h, the pseudovirus-containing culture supernatants were collected and used to transduce PK-15 cells in the presence of polybrene (8 µg/mL). Recombinant PK-15 cells were selected through puromycin treatment (3 µg/mL) for 7 days. Finally, the expression of pB125R in these cells was verified by western blotting and confocal microscopy.

### Reagents and antibodies

Recombinant human TNF-α (300-01A) and IFN-β (300-02BC) were purchased from PeproTech (Cranbury, NJ, USA). SBI-0206965 (HY-16966) and NH_4_Cl (HY-Y1269) were sourced from MedChemExpress (NJ, USA). MG132 (T2154) and 3-methyladenine (T1879) were obtained from TargetMol (Boston, USA). Dimethyl sulfoxide (D8371) was acquired from Solarbio. Protein A/G magnetic beads (PB101-02) were obtained from Vazyme (Nanjing, China).

Rabbit monoclonal antibodies (MAbs) against hemagglutinin (HA) (66006-2-Ig) and glutathione S-transferase (GST) (10000-0-AP) were also obtained from Proteintech (Chicago, IL, USA). The STAT1 rabbit MAb (14994), phospho-STAT1 rabbit MAb (9167), STAT2 rabbit MAb (72604), phospho-STAT2 rabbit MAb (88410), and ATG7 rabbit MAb (8558T) were purchased from Cell Signaling Technology (Danvers, MA, USA). The anti-SQSTM1/P62 rabbit MAb (ab109012) was acquired from Abcam (Shanghai, China). IFNAR2 rabbit polyclonal antibodies (A1769), β-actin rabbit MAb (high dilution) (AC026), and LC3B rabbit MAb (A19665) were obtained from ABclonal (Wuhan, China). Rabbit IgG (A7016) was purchased from Beyotime (Shanghai, China). Mouse Mab against Flag (66008-4-Ig) and MYC (60003-2-Ig) were obtained from Proteintech. Mouse IgG (A7028) was also obtained from Beyotime. HRP-conjugated goat anti-rabbit IgG (SA00001-2) and HRP-conjugated goat anti-mouse IgG (SA00001-1) were obtained from Proteintech. DyLight 488-conjugated goat anti-mouse IgG (A23210) and DyLight 594-conjugated goat anti-rabbit IgG (A23420) were procured from AbbKine (Wuhan, China).

### Constructs

Plasmids encoding HA-, Flag-, Myc-, or GFP-tagged IFNAR1, IFNAR2, JAK1, TYK2, STAT1, STAT2, LC3, β-actin, and pB125R, along with their mutants, were generated by standard molecular biology techniques. The plasmids pSTAT1/2-Fluc, pNF-κB-Fluc, pRL-TK, psPAX2, and pMD2.0G and the empty vector pLOV were kindly provided by Prof. Hong-Bing Shu from Wuhan University. Organelle marker plasmids, including pDsRed2-ER, pDsRed2-Golgi, pDsRed2-Mito, and pDsRed2-LAMP1, which encode proteins that target the endoplasmic reticulum (ER), Golgi, mitochondria, and lysosomes, respectively, were purchased from Clontech. The primers used in this study are listed in Table 1.

**Table 1:** **Primers used in this study**

Plasmids	Primers (5′-3′)
Flag-pB125R Flag-pB125R-N Flag-pB125R-C	F: GCCAAGGGGTCGACCATGGCGGTTTATGCGAAGGA R: TGCTCGAGCGGCCGCTCTAGACTCTAAAAATTTAC F: CCGGGATTTGGATCCATGGCGGTTTATGCG R: CTTGTAGTCGCTAGCGGCATAGTATATGTC F: CCGGGATTTGGATCCATGATCATAAAAAGC R: CCTTGTAGTCGCTAGCTCTAGACTCTAAAAATT

### Dual-luciferase reporter assays

HEK293T cells were transfected with the reporter plasmids pSTAT1/2-Fluc/Rluc (0.01 µg/well) and pRL-TK (0.01 µg/well), along with Flag-pB125R or pRK (10–40 ng/well), by the standard polyethyleneimine (PEI; Polysciences, USA) method. At 20 h post-transfection, the cells were treated with IFN-β for 10 h and then lysed in passive lysis buffer. Luciferase activity was measured via a dual-specific luciferase assay kit (Promega, Madison, WI, USA). *Firefly* luciferase activity was normalized to *Renilla* luciferase activity. The data represent the means from triplicate experiments.

### Reverse transcription‒quantitative PCR (RT‒qPCR)

Total RNA was extracted via TRIzol (TaKaRa Bio, Beijing, China) and reverse-transcribed into cDNA via a HiScript III 1st Strand cDNA Synthesis Kit (Vazyme) following the manufacturer’s instructions. qPCR was performed in triplicate via HiScript II Q RT SuperMix (Vazyme) according to the manufacturer’s protocol. The data are shown as the relative mRNA abundance normalized to that of GAPDH. The primers used for RT‒qPCR are listed in Table 2.

**Table 2:** **Primers used in the RT‒qPCR analysis**

Gene name	Sequence (5′-3′)	
	Forward primer	Reverse primer
hum-*GAPDH*	GACAAGCTTCCCGTTCTCAG	GAGTCAACGGATTTGGTCGT
hum-*STAT1*	GTGGAAAGACAGCCCTGCAT	ACTGGACCCCTGTCTTCAAGAC
hum-*IRF7*	CCCCCATCTTCGACTTCAGA	CAGGACCAGGCTCTTCTCCTT
hum-*ISG56*	TCATCAGGTCAAGGATAGTC	CCACACTGTATTTGGTGTCTAGG
hum-*IL-8*	GAGAGTGATTGAGAGTGGACCAC	CACAACCCTCTGCACCCAGTTT
hum-*TNF-α*	GCCGCATCGCCGTCTCCTAC	CCTCAGCCCCCTCTGGGGTC
sus-*GAPDH*	ACATGGCCTCCAAGGAGTAAGA	GATCGAGTTGGGGCTGTGACT
sus-*STAT1*	GCCTCTCATTGTCACCGAAGAAC	TGGCTGACGTTGGAGATCACCA
sus-*IRF7*	ACATGATCAACCCCGAGCTG	CTCACCAGTATGTGCTGCCA
sus-*IL-8*	TGGCAGTTTTCCTGCTTTCT	CAGTGGGGTCCACTCTCAAT
sus-*TNF-α*	GCCCAAGGACTCAGATCATC	GGCATTGGCATACCCACTCT

### Western blotting

For western blotting, proteins were denatured by 2 × Laemmli sodium dodecyl sulfate‒polyacrylamide gel electrophoresis (SDS‒PAGE) buffer with 2-mercaptoethanol (Sigma‒Aldrich, Germany) and heated at 95 °C for 30 min. Samples were separated via SDS‒PAGE, transferred to polyvinylidene difluoride (PVDF) membranes, and blocked with 5% nonfat milk for 1 h. Membranes were incubated with primary antibodies for 1 h at room temperature, washed six times with wash buffer (TBS with 0.1% Tween-20), and then incubated with horseradish peroxidase (HRP)-conjugated secondary antibodies for 1 h at room temperature. Proteins were visualized via enhanced chemiluminescence (Epizyme, USA).

### Coimmunoprecipitation analysis (Co-IP)

Cells transfected with plasmids expressing specific proteins were lysed in M2 lysis buffer (20 mM Tris–HCl [pH 7.5], 0.5% NP-40, 10 mM NaCl, 3 mM EDTA, and 3 mM EGTA) containing protease inhibitors, followed by sonication for 2.5 min. Lysates were centrifuged at 12 000 × *g* for 10 min at 4 °C. The supernatants were subjected to immunoprecipitation with specific antibodies or anti-Flag M2 magnetic beads (Sigma‒Aldrich) for 4 h. After three washes with cold M2 lysis buffer, the bound proteins were separated by SDS‒PAGE and analysed by western blotting.

### GST pull-down assay

GST pull-down assays were performed following a previously established protocol [30]. To express pB125R in *Escherichia coli*, the recombinant plasmid pGEX-6p-1-pB125R was transformed into *E. coli* BL21 cells. Cells with an optical density (OD) of 600 nm between 0.4 and 0.6 were treated with 0.7 mM isopropyl β-D-thiogalactoside (IPTG; TaKaRa Bio) and cultured at 20 °C for 18 h. The bacterial cultures were centrifuged, and the pellets were resuspended and lysed using a high-pressure homogenizer. The supernatants containing recombinant GST or GST-pB125R were purified using ChromoTek GST-Trap agarose beads (Proteintech). GST and GST-pB125R were incubated with lysates of HEK293T cells expressing ectopic IFNAR2 at 4 °C for 12 h, followed by western blotting using the indicated antibodies.

### Confocal microscopy

Confocal microscopy was performed as previously described [31]. Briefly, PK-15 cells were fixed with 4% paraformaldehyde (Biosharp, Beijing, China) 24 h post-transfection and permeabilized with 0.1% Triton X-100 (Solarbio) for 15 min. The cells were then blocked with 5% bovine serum albumin (BSA; Solarbio) for 30 min and stained with DAPI (Solarbio) or antibodies against Flag and HA. Images were captured with a Zeiss confocal microscope using a 63 × oil objective.

### RNA interference (RNAi)

Small interfering RNAs (siRNAs) targeting ATG7 and control siRNAs (siNCs) were synthesized by GenePharma, Inc. (Shanghai, China). HEK293T cells were transfected with siRNAs using Lipofectamine RNAiMAX Transfection Reagent (Invitrogen, Carlsbad, CA, USA) according to the manufacturer’s instructions. At 48 h post-transfection, the cells were lysed with 2 × SDS‒PAGE sample loading buffer and denatured at 95 °C for 30 min, followed by western blotting with anti-ATG7 antibodies. The target sequences of the ATG7 mRNAs are listed in Table 3.

**Table 3:** **Target sequences for** ***ATG7*** **mRNA**

siRNA name	Sequence (5′-3′)
siRNA#1 siRNA#2	CAGACAAGAAGCUCCUUCUTT CAGCCUGGCAUUUGAUAAATT

### Statistical analysis

Statistical analyses were performed using GraphPad Prism software. The quantitative data are presented as the means ± standard deviations (SD). An unpaired Student’s *t* test was used for data analysis. Statistical significance was set at *P* < 0.05. Asterisks in the figures indicate statistical significance: *P < 0.05, **P < 0.01, ***P < 0.001.

## Results

### Biological characteristics of pB125R

**Figure 1:**
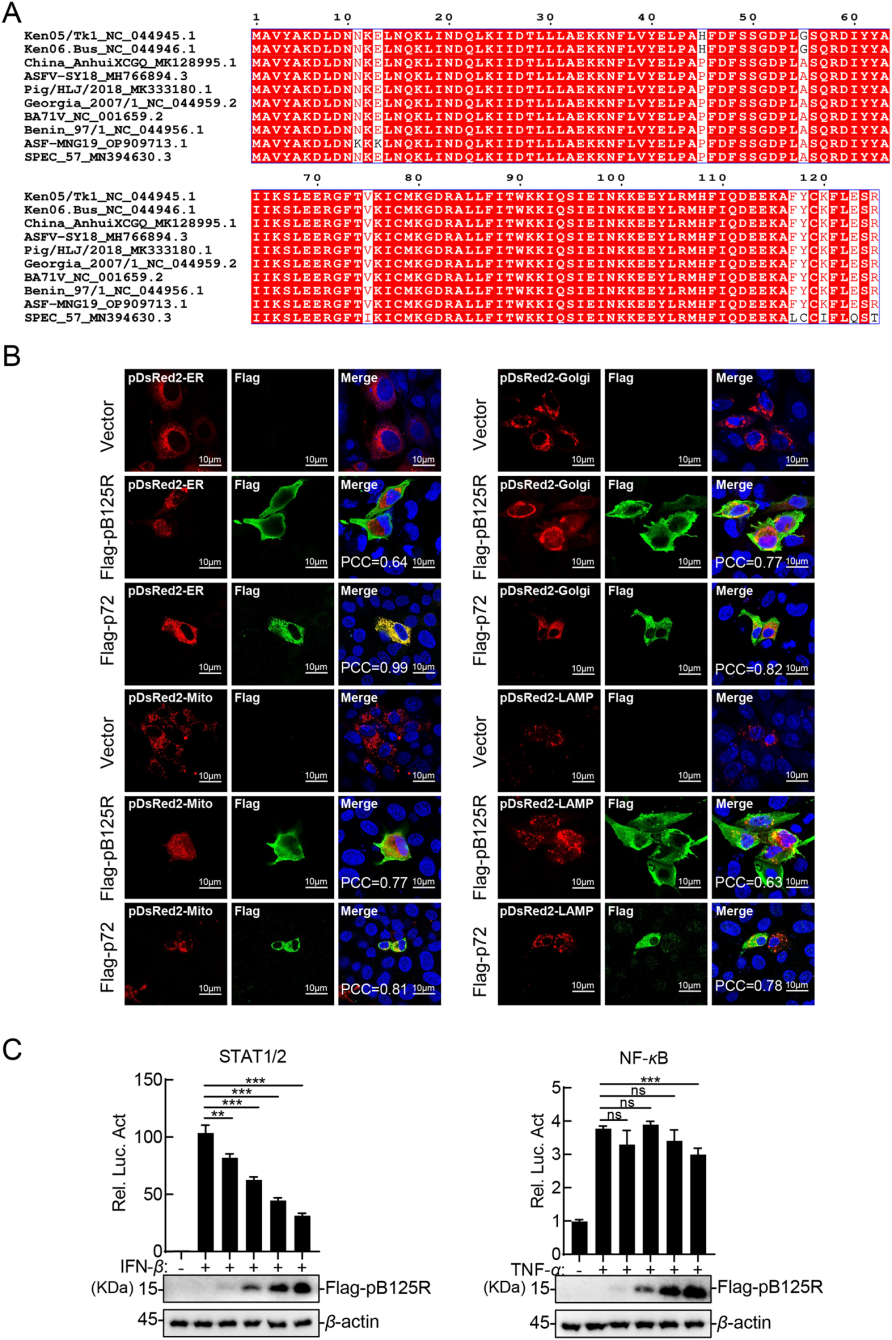
**Biological characteristics of pB125R. A** pB125R is highly conserved among ASFV strains. Multiple sequence alignment of pB125R from different strains was performed using ClustalX 2.1 and visualized with ESPript 3.0. Residues conserved in all sequences are shown in white on a red background. **B** Subcellular localization of pB125R. PK-15 cells were transfected with the empty vector (0.5 µg), Flag-pB125R (0.5 µg) and Flag-p72 (0.5 µg) and organelle markers (pDsRed2-ER, pDsRed2-Golgi, pDsRed2-Mito, or pDsRed2-LAMP1, 0.5 µg each). At 24 h post-transfection, the cells were fixed, permeabilized, and incubated with an anti-Flag antibody and then with a secondary antibody conjugated with AF488 (green). Nuclei were counterstained with DAPI (blue). Organelle markers are visualized in red. Localization was analysed via confocal microscopy. Pearson’s correlation coefficient (PCC) was used to indicate the colocalization between pDsRed2-ER, pDsRed2-Golgi, pDsRed2-Mito, or pDsRed2-LAMP1 (red) and Flag-pB125R or Flag-p72 (green). Scale bar = 10 μm. **C** pB125R inhibited IFN-β-triggered activation of the STAT1/2 promoter in a dose-dependent manner. HEK293T cells were transfected with the Flag-pB125R plasmid at different concentrations (0, 0.01, 0.02, 0.04, or 0.08 µg) along with pSTAT1/2-Fluc (0.01 µg), pNF-κB-Fluc (0.1 µg) or pRL-TK (0.01 µg). Reporter assays and immunoblotting analysis were performed after treatment with IFN-β (10 ng/mL) or TNF-α (10 ng/mL) for 10 h. The data are presented as the means ± SD from one representative experiment performed in triplicate. *P < 0.05; **P < 0.01; ***P < 0.001 (unpaired t test).

The B125R protein (pB125R), a late-transcribed viral protein with 125 amino acids, has an unclear biological function. Structural analysis using SMART and InterPro indicated that pB125R lacks specific functional domains. To evaluate its genetic diversity, we aligned 10 representative sequences from Asian, African, and European pathogenic strains using ClustalX 2.1. The alignment revealed that pB125R is highly conserved across different virus strains (Figure 1A). Subcellular localization was assessed via confocal microscopy by cotransfecting pB125R- or p72-expressing plasmids with organelle-specific markers into PK-15 cells. The distribution of p72, the major capsid protein of ASFV, was examined as a positive control. p72 is localized mainly in the cytoplasm during viral infection [32]. Consistently, our results demonstrated that ectopically expressed p72 was predominantly distributed in the cytoplasm and colocalized with ER markers. Furthermore, partial colocalization of p72 with markers of the Golgi apparatus, mitochondria, and lysosomes was observed. pB125R was predominantly localized to the plasma membrane and partially localized to the endoplasmic reticulum, Golgi apparatus, mitochondria, and lysosomes (Figure 1B). Notably, pB125R significantly increased the lysosomal fluorescence volume, suggesting a functional role. In addition, the results of the reporter assays indicated that pB125R inhibited IFN-β-triggered STAT1/2 promoter activation in a dose-dependent manner but had no effect on TNF-α-induced NF-κB promoter activation (Figure 1C). Taken together, these findings suggest that pB125R may be a membrane-associated protein involved in regulating the host interferon response.

### pB125R inhibits the IFN-β-induced activation of JAK-STAT signalling

**Figure 2:**
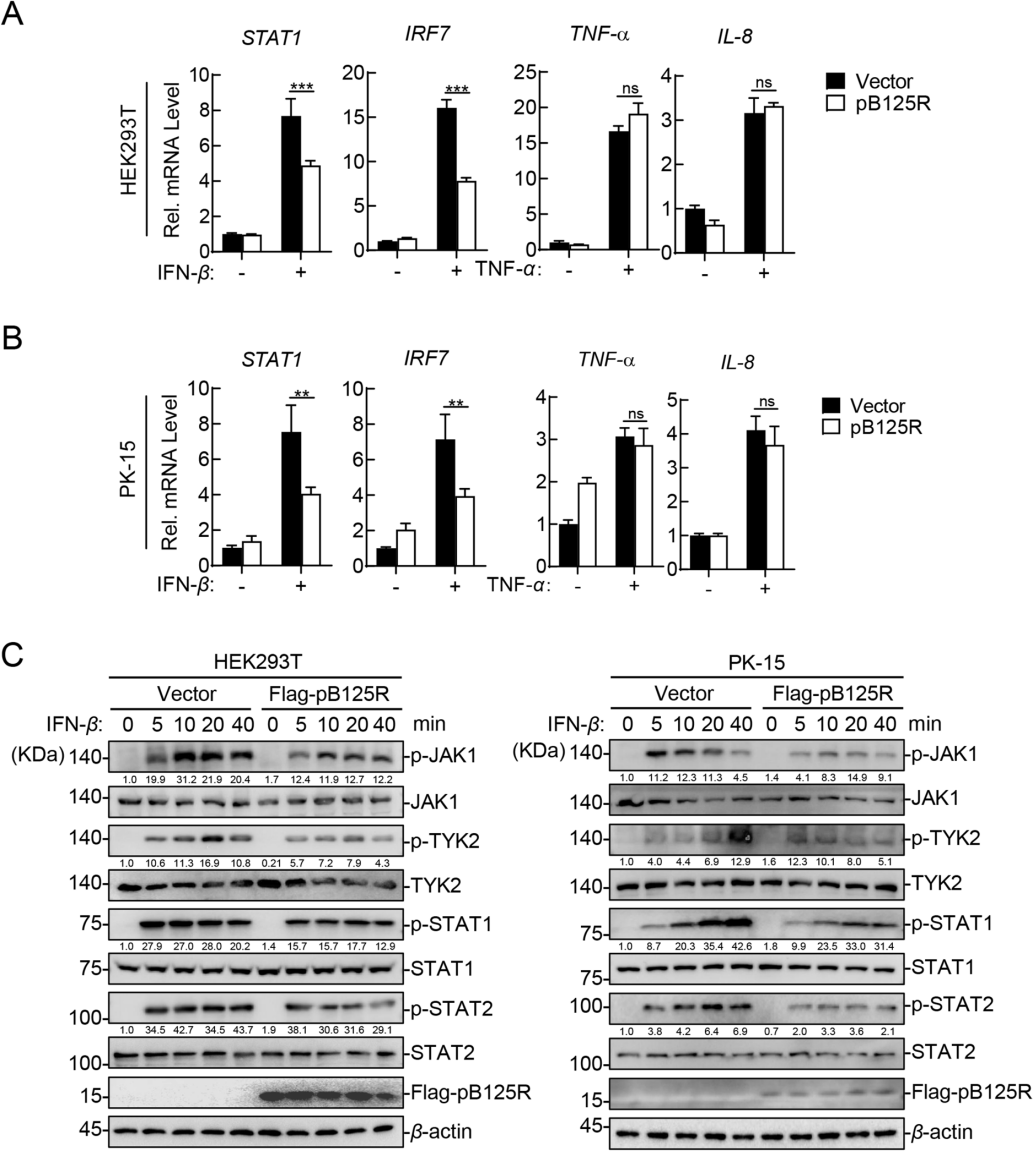
**pB125R inhibits IFN-β-triggered JAK-STAT signalling. A** and** B** pB125R inhibits the IFN-β-triggered transcription of antiviral genes in HEK293T and PK-15 cells. HEK293T cells were transfected with either vector or Flag-pB125R (0.5 µg), or PK-15 cells (stably expressing Flag-pB125R) were treated with IFN-β (10 ng/mL) or TNF-α (10 ng/mL) for 12 h, followed by RT‒qPCR analysis of the *STAT1*, *IRF7*, *TNF‒α* and *IL‒8* genes. The data are presented as the means ± SD from one representative experiment performed in triplicate (A and B). *P < 0.05; **P < 0.01; ***P < 0.001 (unpaired t test). **C** pB125R inhibits the phosphorylation of downstream signalling molecules in the JAK-STAT pathway triggered by IFN-β. HEK293T cells were transfected with either the vector or Flag-pB125R (0.5 µg), or PK-15 cells stably expressing Flag-pB125R or the vector were treated with IFN-β (20 ng/mL) for the indicated times (0, 5, 10, 20, or 40 min). Western blotting was performed using the indicated antibodies. Densitometric analysis of protein expression levels was performed with ImageJ software (target protein/β-actin ratio). The data presented herein are representative of three experiments with analogous results.

To investigate the involvement of pB125R in endogenous JAK-STAT signalling regulation, we measured the transcription levels of *STAT1* and *IRF7* in pB125R-overexpressing HEK293T and PK-15 cells treated with IFN-β. The RT‒qPCR results revealed that pB125R significantly inhibited IFN-β-induced *ISG* mRNA expression but had no effect on TNF-α-induced proinflammatory cytokine transcription (Figures 2A and B). The phosphorylation and activation of JAK1, TYK2, STAT1 and STAT2 are preconditions for JAK-STAT signal transduction; thus, we further investigated the effect of pB125R on the IFN-β-triggered phosphorylation of these molecules in the JAK-STAT signalling pathway. Western blotting revealed that IFN-β significantly induced the phosphorylation of JAK1, TYK2, STAT1 and STAT2 in HEK293T cells and PK-15 cells, whereas the IFN-β-induced phosphorylation of JAK1, TYK2, STAT1, and STAT2 was markedly inhibited in pB125R-overexpressing HEK293T cells (Figure 2C). Similarly, pB125R reduced the phosphorylation of these proteins in PK-15 cells (Figure 2C). These results demonstrate that pB125R negatively regulates the production of IFN-β-induced antiviral genes in both HEK293T and PK-15 cells and that pB125R can specifically inhibit the JAK-STAT signalling pathway triggered by IFN-β. In addition, pB125R counteracts host antiviral responses at the initial step of the JAK-STAT signalling pathway, and IFNAR1/IFNAR2 may be a possible target of pB125R.

### pB125R interacts with IFNAR2

**Figure 3:**
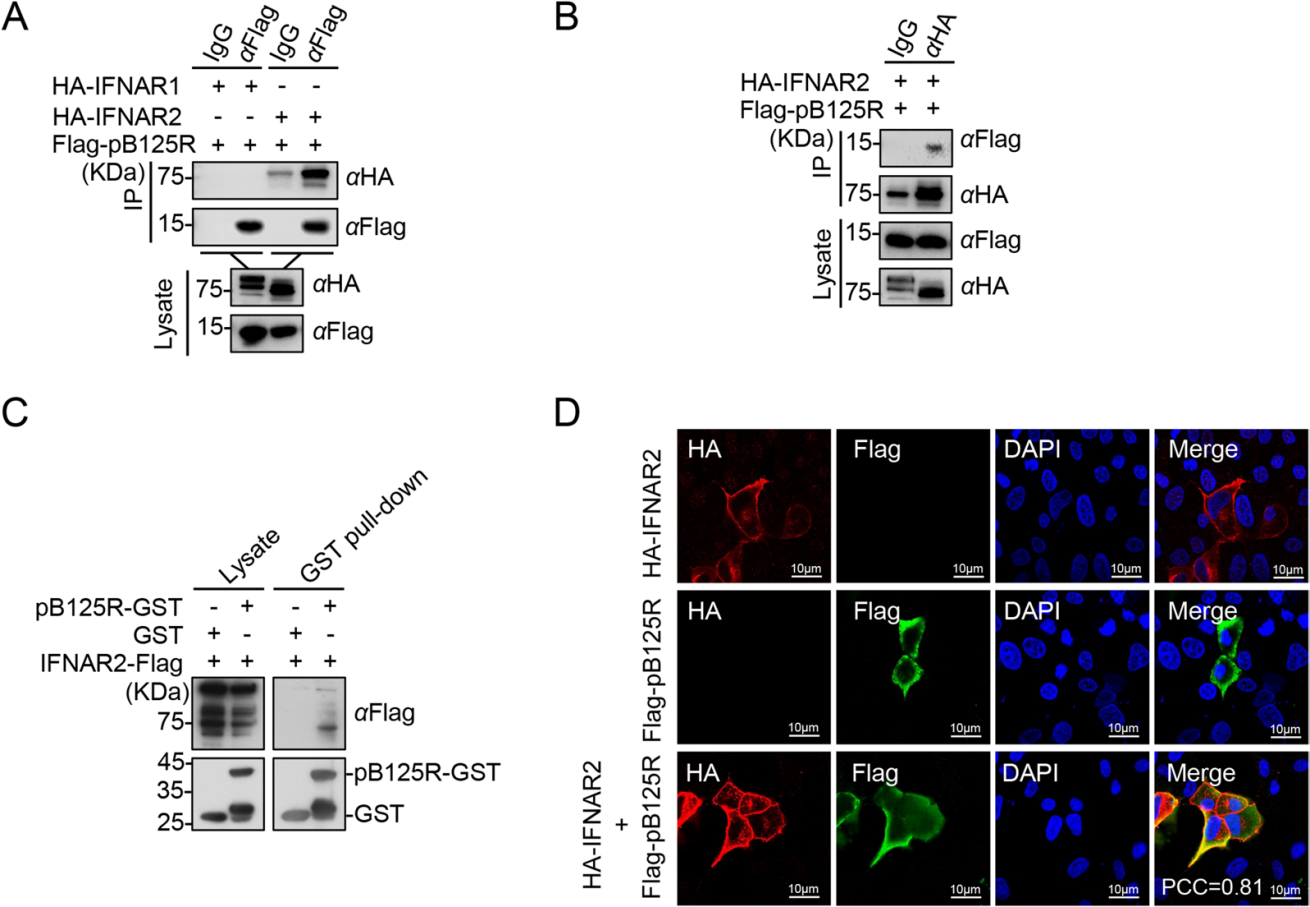
**pB125R interacts with IFNAR2. A** and** B** pB125R interacts with IFNAR2. HEK293T cells were transfected with plasmids expressing Flag-pB125R (5 µg) and HA-IFNAR1 (3 µg) or HA-IFNAR2 (3 µg) for 24 h and then lysed for coimmunoprecipitation with IgG, anti-Flag or anti-HA, followed by immunoblotting analysis with the indicated antibodies. **C** pB125R binds to IFNAR2 in vitro. GST or the recombinant GST-tagged pB125R was incubated with Flag-IFNAR2 for 8 h, followed by immunoblotting analysis with the indicated antibodies. **D** pB125R and IFNAR2 colocalize at the cell membrane. PK-15 cells stably expressing ASFV pB125R were transfected with plasmids expressing HA-IFNAR2 (1 µg) for 24 h, after which the cells were fixed for immunostaining before being subjected to confocal microscopy. The PCC was used to indicate colocalization between HA-IFNAR2 (red) and Flag-pB125R (green). Scale bar = 10 μm. The data presented herein are representative of three experiments with analogous results (**A**–**D**).

Given that pB125R suppressed all the tested phosphorylation events of JAK-STAT signalling, we hypothesized that it might function at the IFNAR1 or IFNAR2 level. Transient transfection and coimmunoprecipitation (co-IP) experiments confirmed that pB125R interacted with IFNAR2 but not with IFNAR1 (Figures 3A, B). GST pull-down assays revealed direct binding between pB125R and IFNAR2 (Figure 3C). Confocal microscopy revealed that the colocalization of pB125R and IFNAR2 occurred primarily at the plasma membrane, with a PCC of 0.81 (Figure 3D). These results show that pB125R mainly targets IFNAR2 to negatively control the ASFV-triggered IFN response.

### pB125R promotes the degradation of IFNAR2

**Figure 4:**
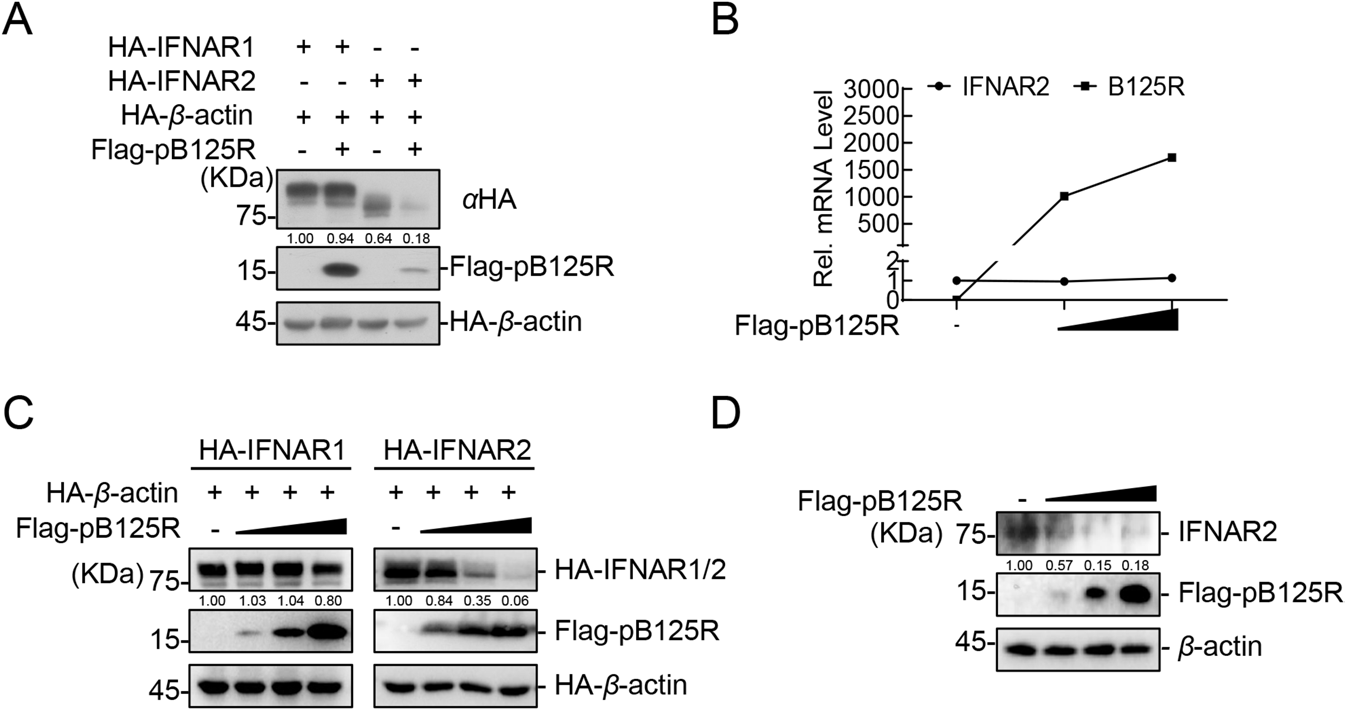
**pB125R promotes IFNAR2 degradation. A** pB125R specifically inhibits IFNAR2 expression. HEK293T cells were transfected with plasmids expressing HA-β-actin (0.1 µg), Flag-pB125R (0.5 µg), HA-IFNAR1 (0.1 µg) or HA-IFNAR2 (0.05 µg) for 24 h, followed by western blotting analysis with the indicated antibodies. **B** pB125R has no effect on the transcription of *IFNAR2* HEK293T cells were transfected with HA-IFNAR2 (0.05 µg) and either the Flag-pB125R expression plasmid or the HA-IFNAR2 expression plasmid (0, 0.2, or 0.4 µg) for 24 h, and total RNA was extracted for RT‒qPCR analysis of the *IFNAR2* and *B125R* genes. The data are presented as the means ± SD from one representative experiment performed in triplicate. **C** pB125R reduces the protein level of IFNAR2 in a dose-dependent manner. HEK293T cells were transfected with plasmids expressing HA-β-actin (0.1 µg), various concentrations (0, 0.1, 0.2, or 0.4 µg) of Flag-pB125R, HA-IFNAR1 (0.1 µg) or HA-IFNAR2 (0.05 µg) for 24 h, followed by western blotting analysis with the indicated antibodies. **D** pB125R reduced endogenous IFNAR2 protein levels in a dose-dependent manner. HEK293T cells were transfected with different concentrations (0, 0.1, 0.2, or 0.4 µg) of the Flag-pB125R expression plasmid. Twenty-four hours later, western blotting analysis was performed using the indicated antibodies. Densitometric analysis of protein expression levels was performed with ImageJ software (target protein/β-actin ratio) (**A**, **C**, and **D**). The data presented herein are representative of three experiments with analogous results (**A**, **C**, and **D**).

To elucidate how pB125R targets IFNAR2, HEK293T cells were co-transfected with pB125R and IFNAR1/2. Western blotting revealed that pB125R significantly inhibited IFNAR2 expression but had no effect on IFNAR1 (Figure 4A). The RT‒qPCR results confirmed that pB125R did not affect IFNAR2 transcription (Figure 4B). Further experiments indicated that pB125R reduced IFNAR2 levels (Figure 4C) and decreased endogenous IFNAR2 protein levels in a dose-dependent manner (Figure 4D). Thus, pB125R may reduce the protein level of IFNAR2 by promoting its degradation.

### pB125R degrades IFNAR2 via autophagy

**Figure 5:**
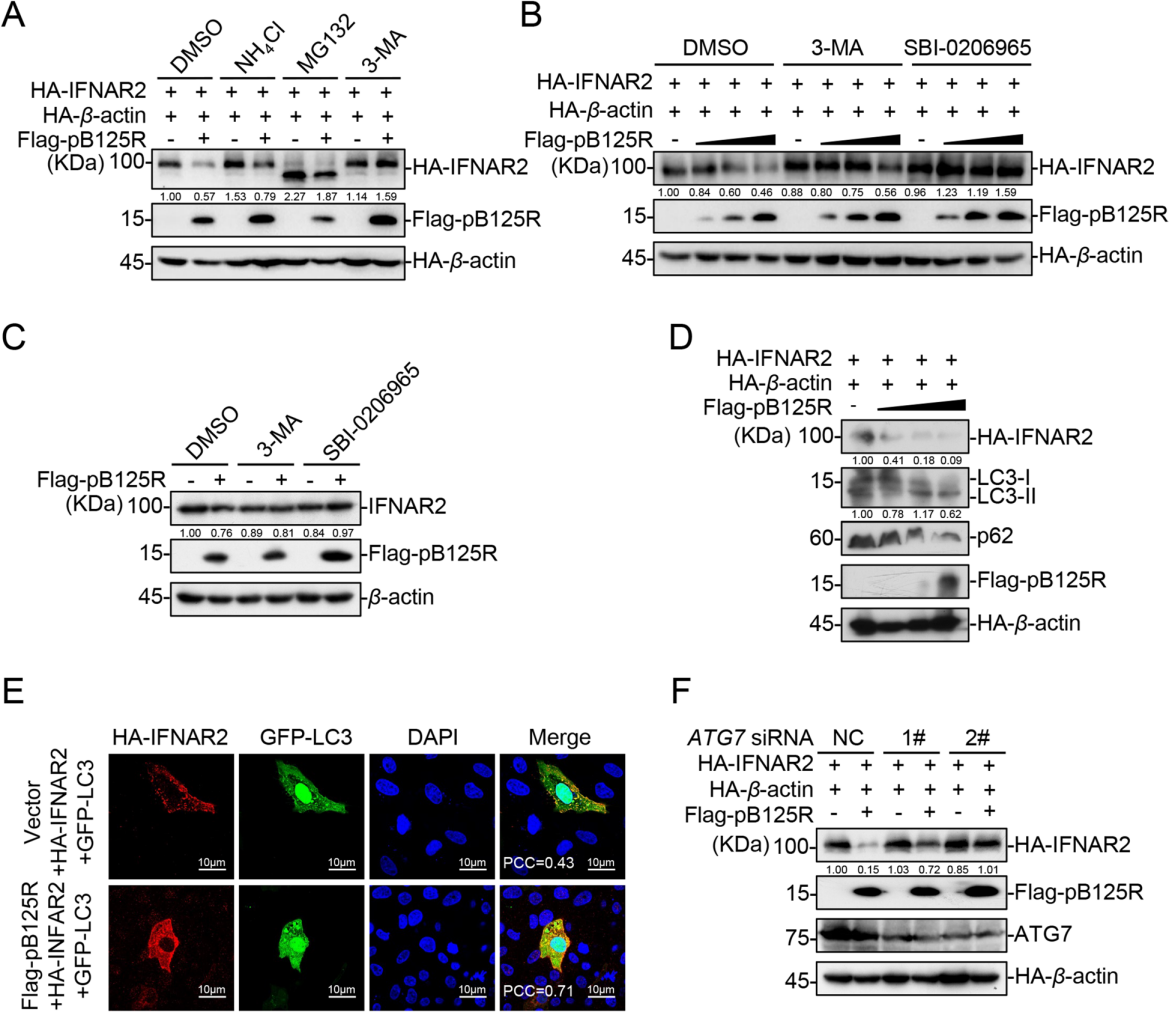
**pB125R degrades IFNAR2 via autophagy. A** Degradation of IFNAR2 by pB125R is inhibited by the autophagy inhibitor 3-MA. HEK293T cells transfected with the indicated plasmids were treated with DMSO, NH4Cl (20 mM), MG132 (20 µM), or 3-MA (1 mM) for 8 h before immunoblot analysis. **B** Both 3-MA and SBI-0206965 inhibited pB125R-induced IFNAR2 degradation. HEK293T cells were transfected with HA-IFNAR2 (0.05 µg), HA-β-actin (0.1 µg), and Flag-pB125R (0, 0.1, 0.2, or 0.4 µg) and then treated with DMSO, 3-MA (1 mM), or SBI-0206965 (20 µM) for 8 h before immunoblotting. **C** Both 3-MA and SBI-0206965 completely inhibited pB125R-induced endogenous IFNAR2 degradation. HEK293T cells transfected with the indicated plasmids were treated with DMSO, 3-MA (1 mM) or SBI-0206965 (20 µM) for 8 h before immunoblot analysis. **D** pB125R promotes autophagy activation. HEK293T cells were transfected with HA-IFNAR2 (0.05 µg), HA-β-actin (0.1 µg), and Flag-pB125R (0, 0.1, 0.2, or 0.4 µg), followed by immunoblotting analysis. **E** pB125R promotes the formation of LC3 spots. PK-15 cells transfected with the indicated plasmids were detected with a confocal laser scanning microscope. The colocalization of GFP-LC3 (green) and HA-IFNAR2 (red) was analysed using the Coloc2 tool of ImageJ/FIJI and is shown as the PCC. Scale bar = 10 μm.** F** Knockdown of ATG7 inhibits pB125R-mediated degradation of IFNAR2. The control siRNAs (NC) or siRNAs targeting ATG7, denoted #1 and #2, were transfected into HEK293T cells for 24 h, followed by transfection with HA-IFNAR2 (0.05 µg), HA-β-actin (0.1 µg), and Flag-pB125R (0.5 µg) or the vector for 24 h. Next, immunoblotting analysis with the indicated antibodies was performed. Densitometric analysis of protein expression levels was performed with ImageJ software (target protein/β-actin ratio) (**A**–**D**, and **F**). The data presented herein are representative of three experiments with analogous results (**A**–**F**).

Degradation is one of the primary strategies for regulating protein function. At least three systems are involved in protein degradation: the ubiquitin‒proteasome, lysosome, and autophagosome pathways. To investigate the pathway through which pB125R promotes IFNAR2 degradation, we utilized inhibitors of various degradation pathways. Our results showed that pB125R-mediated degradation of IFNAR2 was completely inhibited by the autophagy inhibitor 3-MA but not by the proteasome inhibitor MG132 or the lysosomal inhibitor NH_4_Cl (Figure 5A). Additionally, the autophagy inhibitor SBI-0206965 effectively inhibited pB125R-induced IFNAR2 degradation (Figures 5B and C), confirming that pB125R degrades IFNAR2 via autophagy. We also examined the expression of LC3-II and p62 and found that pB125R overexpression significantly increased LC3-II levels while promoting the degradation of IFNAR2 and p62, indicating that pB125R triggers cellular autophagy (Figure 5D). Furthermore, pB125R enhanced LC3-GFP spot formation and colocalization with IFNAR2 in PK-15 cells (Figure 5E). In ATG7-knockdown cells, pB125R-mediated degradation of IFNAR2 was restored (Figure 5F). These findings suggest that pB125R induced cellular autophagy and mediated the degradation of IFNAR2 via the autophagy pathway. Inhibition of cellular autophagy effectively counteracted pB125R-mediated IFNAR2 degradation.

### Identification of pB125R-interacting host factors involved in autophagy modulation

**Figure 6:**
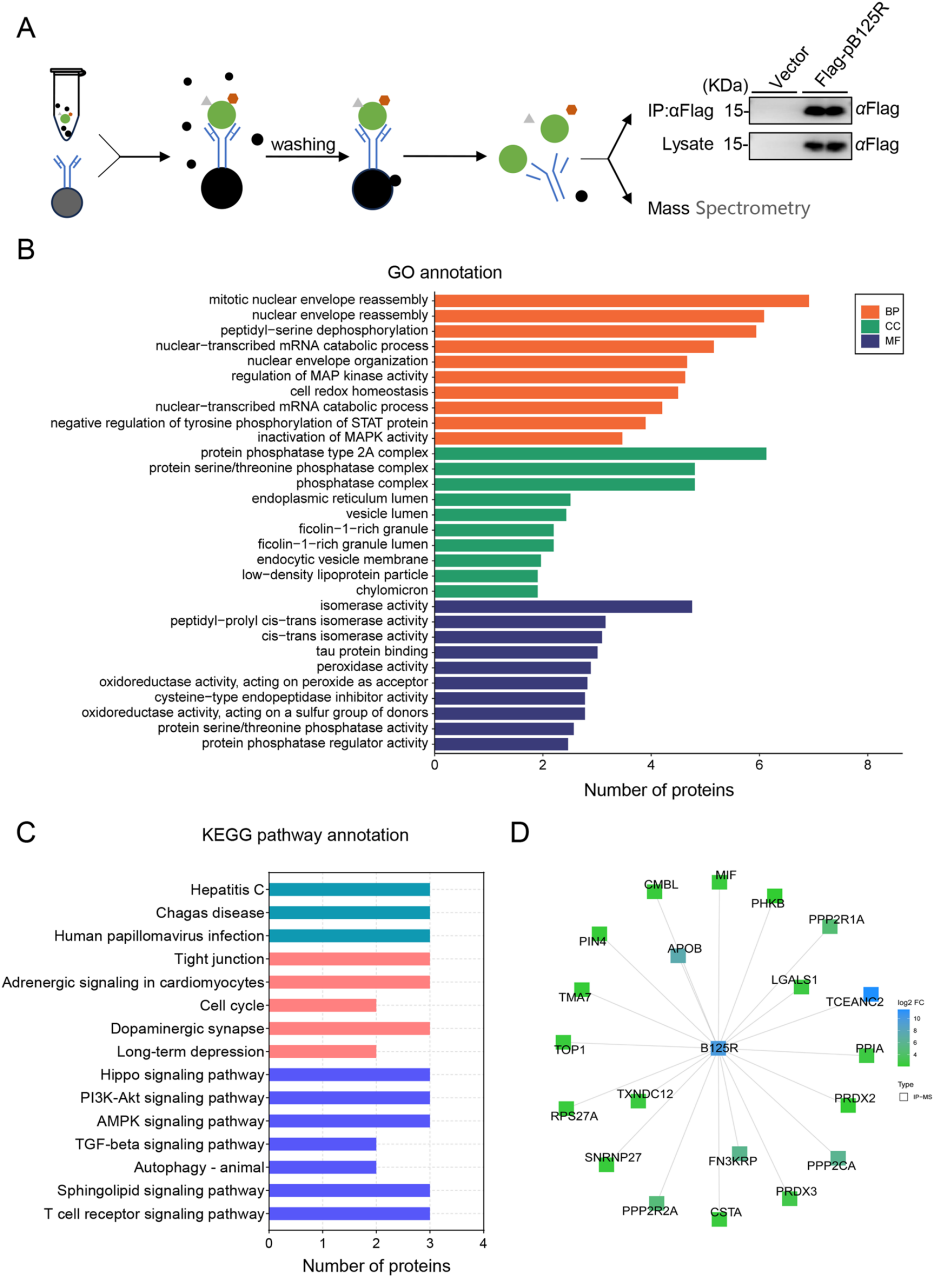
**Proteomic analysis of the pB125R-host interactome. A** HEK293T cells were transfected with Flag-pB125R (5 µg) for 24 h, lysed for co-IP with anti-Flag MAb, and subjected to western blot analysis with the indicated antibodies. High-throughput proteomic analysis was performed on immunoprecipitated samples via mass spectrometry (MS), with two replicates of each group. **B** and **C**, KEGG pathway analysis and GO category functional enrichment were performed for the 20 identified proteins. **D** The protein‒protein interaction network of proteins interacting with pB125R was analysed via the STRING database.

pB125R promotes the degradation of IFNAR2 through the autophagy pathway, whereas how pB125R functions remains unclear. To investigate the host factors through which pB125R regulates IFNAR2 degradation, the empty vector or Flag-pB125R plasmid was transfected into HEK293T cells. Next, immunoprecipitation (IP) experiments and mass spectrometry (MS) analysis were performed with two replicates of each group according to the test manual of SpecAlly. The results showed that pB125R was enriched successfully. Compared with those in the vector group, proteins whose fold change was greater than 4 were considered candidate interactors of pB125R (Figure 6A). A total of 20 host factors that potentially interact with pB125R were identified, among which host molecules involved in the autophagy pathway include the catalytic subunit (encoded by *PPP2CA*), the scaffolding subunit (encoded by *PPP2R1A*), and the regulatory subunit (encoded by *PPP2R2A*) of protein phosphatase 2A (PP2A). In the Gene Ontology (GO) analysis of the IP‒MS results, we observed a significant proportion of molecules associated with the autophagosome system across CC, MF, and BP (Figure 6B). Furthermore, Kyoto Encyclopedia of Genes and Genomes (KEGG) pathway analysis revealed the involvement of multiple signalling pathways, such as the autophagy pathway, the PI3K‒Akt signalling pathway, and the AMPK signalling pathway (Figure 6C). Moreover, protein‒protein interaction analysis demonstrated that pB125R interacts with the catalytic subunit, the scaffolding subunit, and the regulatory subunit of PP2A (Figure 6D). On the basis of the proteomic analysis, we speculated that the PP2A catalytic subunit, scaffolding subunit, and regulatory subunit may be the key host factors involved in pB125R-triggered autophagic degradation of IFNAR2.

### Both terminal regions of pB125R are essential for suppressing JAK-STAT signalling activation

**Figure 7:**
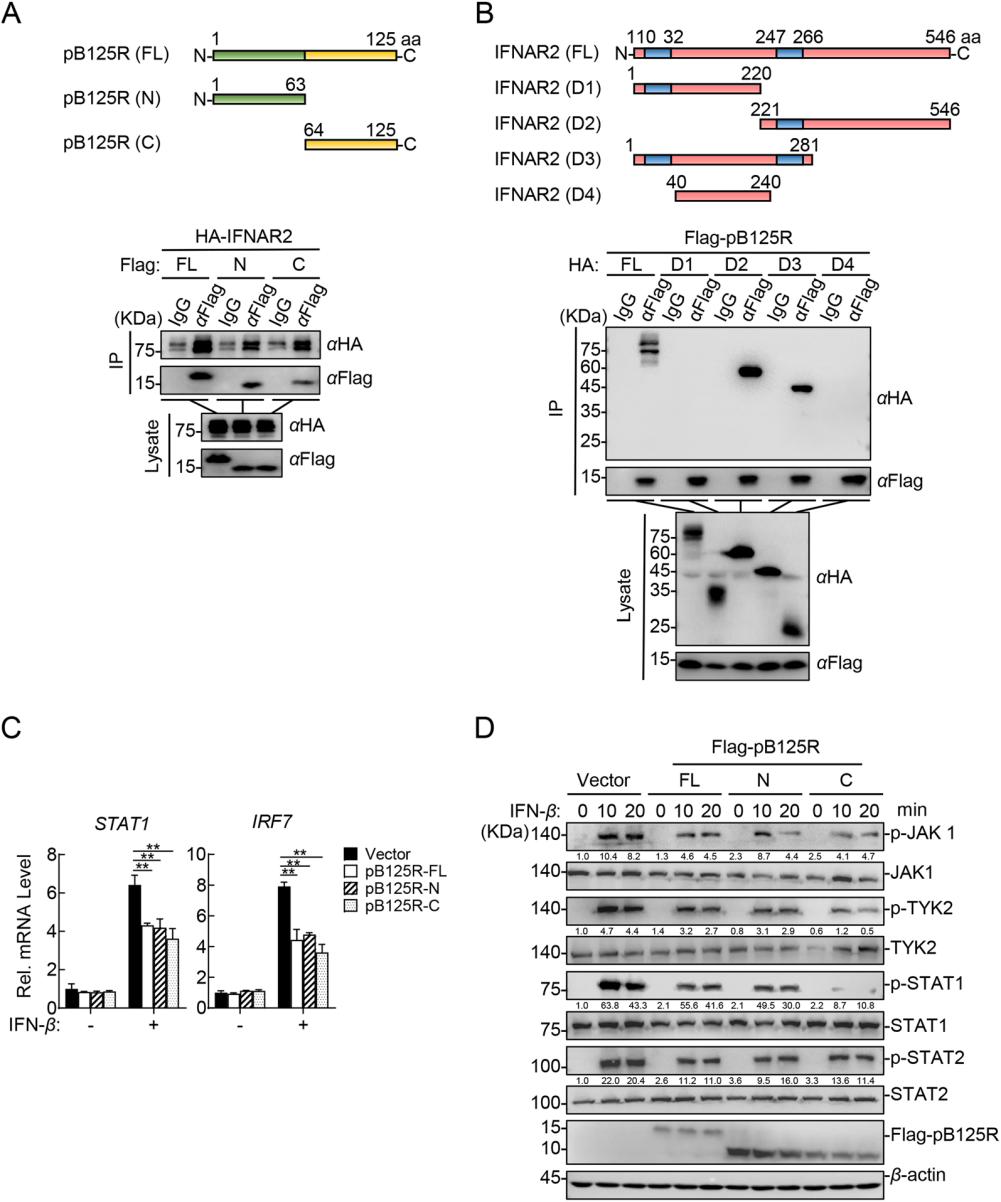
**Both terminal regions of pB125R are essential for suppressing JAK-STAT signalling activation. A** Schematic illustration of the truncated pB125R mutants. The truncation mutant Flag-pB125R-N contains the N-terminal 1–63 aa, whereas Flag-pB125R-C contains the C-terminal 64–125 aa. Plasmids expressing Flag-pB125R-FL (3 µg), Flag-pB125R-N (3 µg), or Flag-pB125R-C (3 µg) were cotransfected into HEK293T cells with the HA-IFNAR2-expressing plasmid (2 µg) for 24 h, followed by co-IP and immunoblotting analysis. **B** Schematic illustration of the truncated IFNAR2 mutants. The truncation mutant IFNAR2-D1 contains the N-terminal 1–220 aa, whereas IFNAR2-D2 contains the C-terminal 221–546 aa, IFNAR2-D3 contains the N-terminal 1–281 aa, and IFNAR2-D4 contains the N-terminal 40–240 aa. Plasmids expressing HA-IFNAR2-FL (2 µg), HA-IFNAR2-D1 (2 µg), HA-IFNAR2-D2 (2 µg), HA-IFNAR2-D3 (2 µg), or HA-IFNAR2-D4 (2 µg) were cotransfected into HEK293T cells with the Flag-pB125R-expressing plasmid (3 µg) for 24 h, followed by co-IP and immunoblotting analysis. **C** pB125R-N and pB125R-C reduced the IFN-β-triggered transcription of *STAT1* and *IRF7*. HEK293T cells were transfected with Flag-pB125R-FL (0.5 µg), Flag-pB125R-N (0.5 µg), Flag-pB125R-C (0.5 µg), or vector (0.5 µg) for 12 h and then treated with IFN-β (10 ng/mL) for 12 h. Total RNA was analysed by RT‒qPCR for the *STAT1* and *IRF7* genes. The data are shown as the means ± SD (*n* = 3). *P < 0.05; **P < 0.01; ***P < 0.001 (unpaired t test). **D** Both the N- and C-termini of pB125R impaired the IFN-β-triggered phosphorylation of JAK-STAT pathway molecules. HEK293T cells transfected with Flag-pB125R-FL (0.5 µg), Flag-pB125R-N (0.5 µg), Flag-pB125R-C (0.5 µg), or vector (0.5 µg) were treated with IFN-β (20 ng/mL) for the indicated times (0, 10, or 20 min), followed by western blot analysis. Densitometric analysis of protein expression levels was performed with ImageJ software (target protein/β-actin ratio). The data presented herein are representative of three experiments with analogous results (**A**–**D**).

To determine the regions of IFNAR2 and pB125R responsible for their interaction and the suppression of JAK-STAT signalling, we constructed plasmids expressing full-length or truncated pB125R (Figure 7A) and IFNAR2 (Figure 7B). Domain mapping indicated that both the N- and C-terminal regions of pB125R interact with IFNAR2 (Figure 7A). The transmembrane domain of IFNAR2 (aa 247–266) was also implicated in its interaction with pB125R (Figure 7B). We evaluated whether the N- and C-terminal regions of pB125R are essential for suppressing JAK-STAT activation. RT‒qPCR revealed that expression of either the terminal region of pB125R dramatically reduced the transcript levels of the *STAT1* and *IRF7* genes in HEK293T cells (Figure 7C). Correspondingly, the phosphorylation of JAK1, TYK2, STAT1, and STAT2 was markedly impaired in HEK293T cells expressing either terminal region (Figure 7D). Taken together, our results suggest that the N- and C-terminal regions of pB125R are essential for inhibiting the activation of the JAK-STAT signalling pathway.

## Discussion

Recent studies have highlighted the strong association between the ability of ASFV to evade the host antiviral immune response and its pathogenicity. Deleting virulence-related genes from ASFV has been suggested as a potential strategy for developing live attenuated vaccines. However, the biological characteristics, including subcellular locations, transcriptional features, and functions, of approximately half of the viral proteins remain poorly understood. This knowledge gap, particularly regarding virion structure and virulence-related genes, has significantly hampered efforts to prevent ASF. However, the role of IFN in ASFV infection remains controversial. Early studies revealed that bovine IFN-α inhibited replication of both the virulent CC83 strain and the attenuated BA71 strain in porcine mononuclear and alveolar macrophages [33]. Human IFN-α also inhibited replication of the BA71V strain in Vero cells [23]. More recent studies have shown that recombinant porcine IFN-α produced in *Escherichia coli* inhibits ASFV SY18 replication both in vitro and in vivo and reduces the viral load in surviving pigs [22]. Conversely, another study reported that recombinant porcine IFN-α did not inhibit replication of the virulent strains BA71, Georgia 2007/1, OUR T88/1, and Pr4 in porcine alveolar macrophages (PAMs) or peripheral blood monocytes, whereas it significantly suppressed replication of the weaker strains OUR T88/3 and Pr4Δ35 [34]. These findings suggest that the efficacy of IFN in ASFV infection may be influenced by factors such as viral virulence, cell type and treatment regimen. However, further studies are needed to elucidate the underlying mechanisms and potential therapeutic applications of IFN-γ in ASFV infection.

In this study, we identified pB125R as a negative regulator of the JAK-STAT signalling pathway and revealed a novel mechanism responsible for ASFV immune evasion. We found that transient transfection of pB125R into HEK293T cells significantly inhibited the IFN-β-induced activation of the STAT1/2 promoter, as well as the transcription of the antiviral genes *STAT1* and *IRF7*. Additionally, it blocked the phosphorylation of key proteins in the JAK-STAT pathway, such as JAK1, TYK2, STAT1, and STAT2. Similar results were observed in PK-15 cells stably expressing pB125R, suggesting that this protein plays a critical role in ASFV immune evasion across different cell types.

Interestingly, pB125R inhibited the phosphorylation of all the tested proteins (JAK1, TYK2, STAT1, and STAT2) in the JAK-STAT pathway triggered by IFN-β. This led us to hypothesize that pB125R might function at the initial stage of the signalling pathway, particularly at the level of the IFNR. Co-IP analysis and GST pull-down assays demonstrated that pB125R directly interacts with IFNAR2, a subunit of the interferon-α/β receptor. Consistent with this finding, confocal microscopy revealed that pB125R colocalizes with IFNAR2 at the plasma membrane, suggesting that pB125R could be a membrane-associated protein, although no transmembrane domain was predicted via bioinformatic tools such as SMART, InterPro, and SMARTBLAST.

In addition, both the N-terminal and C-terminal domains of pB125R were able to bind to IFNAR2 and inhibit JAK-STAT signalling, indicating that these regions are essential for suppressing the ASFV-induced IFN response. This observation suggests that the distinct structural domains of pB125R may function independently or, alternatively, that pB125R may have a single functional structural domain that allows both terminal regions to contribute to its overall function owing to their specific conformation or flexibility [35].

During our experiments, we discovered that pB125R significantly reduced IFNAR2 protein levels without affecting IFNAR2 transcription, indicating that pB125R promotes the degradation of IFNAR2 at the protein level. To elucidate the pathway through which pB125R induces IFNAR2 degradation, we used inhibitors targeting different protein degradation systems. Our results showed that autophagy inhibitors (3-MA and SBI-0206965) were able to restore IFNAR2 levels, whereas proteasome inhibitors (MG132) and lysosomal inhibitors (NH_4_Cl) had no effect. These findings suggest that pB125R mediates IFNAR2 degradation through autophagy, a cellular degradation process that maintains homeostasis by selectively removing damaged organelles and proteins [36].

Despite these findings, the exact mechanism by which IFNAR2 is degraded via autophagy remains unclear. To further explore this, Co-IP and MS were performed to identify host factors that interact with pB125R and may be involved in promoting the autophagy-mediated degradation of IFNAR2. A total of 20 host proteins were identified, including three different types of subunits that make up the protein phosphatase 2A (PP2A) complex. PP2A is a key serine/threonine phosphatase that has been reported to regulate autophagy through multiple signalling pathways. One study reported that PP2A inactivates AKT through dephosphorylation, thereby promoting autophagy by inhibiting AKT/mTOR signalling [37]. Another study reported that the activation of PP2A leads to the dephosphorylation of AMPKα, thereby activating the AMPK signalling pathway and subsequently inducing autophagy [38]. Another study revealed that PP2A activates DAPK3 kinase activity via the dephosphorylation of DAPK3, with activated DAPK3 phosphorylating Beclin1, thereby triggering autophagy [39]. Thus, we speculate that pB125R might activate the autophagy pathway by enhancing PP2A activity, leading to the induction of autophagy. However, further research is necessary to clarify whether and how pB125R interacts with PP2A and promotes the autophagy-mediated degradation of IFNAR2.

**Figure 8:**
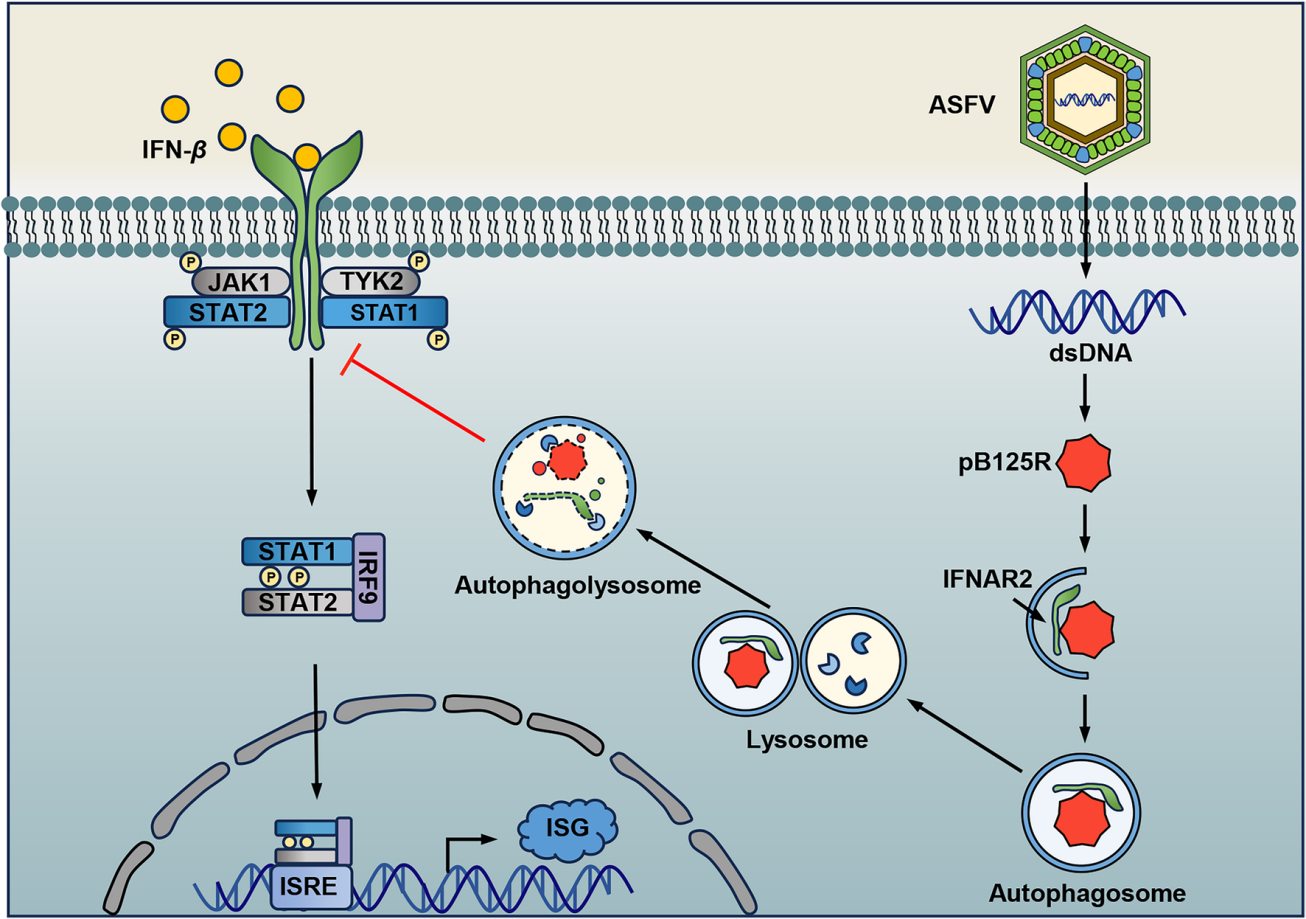
**Schematic diagram of the mechanism by which pB125R suppresses host IFN responses.** ASFV pB125R inhibits IFN-I responses by targeting IFNAR2. It reduces the phosphorylation of downstream signalling molecules in the JAK-STAT pathway by promoting IFNAR2 degradation via autophagy, leading to decreased ISGF3 nuclear translocation and the inhibition of ISG expression.

In conclusion, as illustrated in Figure 8, our findings demonstrate that pB125R plays a crucial role in ASFV immune evasion by regulating the IFN-β-triggered JAK-STAT signalling pathway. This effect is achieved by targeting IFNAR2 and promoting its degradation via autophagy. Previous studies have shown that deleting the pB125R gene from ASFV can attenuate the virulence of recombinant viruses, making pB125R a candidate virulence factor [40]. However, single gene deletions alone may not be sufficient to completely weaken ASFV, as many viral proteins likely function together during infection and pathogenesis. For example, these proteins may contribute to immune evasion at different stages of viral infection. Therefore, further studies on how ASFV proteins interact synergistically are essential for developing effective ASF vaccines.

## Data Availability

All the data generated or analysed during this study are included in this published article.

## References

[1] Juszkiewicz M, Walczak M, Woźniakowski G, Podgórska K (2023) African swine fever: transmission, spread, and control through biosecurity and disinfection, including Polish trends. Viruses 15:2275 38005951 10.3390/v15112275 PMC10674562

[2] Eustace Montgomery R (1921) On a form of swine fever occurring in British East Africa (Kenya Colony). J Comp Pathol Ther 34:159–191

[3] Dixon LK, Stahl K, Jori F, Vial L, Pfeiffer DU (2020) African swine fever epidemiology and control. Annu Rev Anim Biosci 8:221–246 31743062 10.1146/annurev-animal-021419-083741

[4] Teklue T, Sun Y, Abid M, Luo Y, Qiu HJ (2020) Current status and evolving approaches to African swine fever vaccine development. Transbound Emerg Dis 67:529–542 31538406 10.1111/tbed.13364

[5] Gaudreault NN, Madden DW, Wilson WC, Trujillo JD, Richt JA (2020) African Swine fever virus: an emerging DNA arbovirus. Front Vet Sci 7:215 32478103 10.3389/fvets.2020.00215 PMC7237725

[6] Alonso C, Borca M, Dixon L, Revilla Y, Rodriguez F, Escribano JM, Ictv Report C (2018) ICTV virus taxonomy profile: Asfarviridae. J Gen Virol 99:613–614 29565243 10.1099/jgv.0.001049 PMC12662184

[7] Zheng YX, Li S, Li SH, Yu SX, Wang QH, Zhang KH, Qu L, Sun Y, Bi YH, Tang FC, Qiu HJ, Gao GF (2022) Transcriptome profiling in swine macrophages infected with African swine fever virus at single-cell resolution. Proc Natl Acad Sci USA 119:e2201288119 35507870 10.1073/pnas.2201288119 PMC9171760

[8] Akira S, Uematsu S, Takeuchi O (2006) Pathogen recognition and innate immunity. Cell 124:783–801 16497588 10.1016/j.cell.2006.02.015

[9] Randall RE, Goodbourn S (2008) Interferons and viruses: an interplay between induction, signalling, antiviral responses and virus countermeasures. J Gen Virol 89:1–47 18089727 10.1099/vir.0.83391-0

[10] Darnell JE Jr, Kerr IM, Stark GR (1994) Jak-STAT pathways and transcriptional activation in response to IFNs and other extracellular signaling proteins. Science 264:1415–1421 8197455 10.1126/science.8197455

[11] Lukhele S, Boukhaled GM, Brooks DG (2019) Type I interferon signaling, regulation and gene stimulation in chronic virus infection. Semin Immunol 43:101277 31155227 10.1016/j.smim.2019.05.001 PMC8029807

[12] Takaoka A, Yanai H (2006) Interferon signalling network in innate defence. Cell Microbiol 8:907–922 16681834 10.1111/j.1462-5822.2006.00716.x

[13] García-Belmonte R, Pérez-Núñez D, Pittau M, Richt JA, Revilla Y (2019) African swine fever virus Armenia/07 virulent strain controls interferon beta production through the cGAS-STING pathway. J Virol 93:e02298-18 30918080 10.1128/JVI.02298-18 PMC6613762

[14] Liu X, Chen H, Ye G, Liu H, Feng C, Chen W, Hu L, Zhou Q, Zhang Z, Li J, Zhang X, He X, Guan Y, Wu Z, Zhao D, Bu Z, Weng C, Huang L (2024) African swine fever virus pB318L, a trans-geranylgeranyl-diphosphate synthase, negatively regulates cGAS-STING and IFNAR-JAK-STAT signaling pathways. PLoS Pathog 20:e1012136 38620034 10.1371/journal.ppat.1012136 PMC11018288

[15] Li Y, Huang L, Li H, Zhu Y, Yu Z, Zheng X, Weng C, Feng WH (2024) ASFV pA151R negatively regulates type I IFN production via degrading E3 ligase TRAF6. Front Immunol 15:1339510 38449860 10.3389/fimmu.2024.1339510 PMC10914938

[16] Dodantenna N, Cha JW, Chathuranga K, Chathuranga WAG, Weerawardhana A, Ranathunga L, Kim Y, Jheong W, Lee JS (2024) The African swine fever virus virulence determinant DP96R suppresses Type I IFN production targeting IRF3. Int J Mol Sci 25:2099 38396775 10.3390/ijms25042099 PMC10889005

[17] Sunwoo SY, García-Belmonte R, Walczak M, Vigara-Astillero G, Kim DM, Szymankiewicz K, Kochanowski M, Liu L, Tark D, Podgórska K, Revilla Y, Pérez-Núñez D (2024) Deletion of MGF505-2R gene activates the cGAS-STING pathway leading to attenuation and protection against virulent African swine fever virus. Vaccines 12:407 38675789 10.3390/vaccines12040407 PMC11054455

[18] Riera E, Pérez-Núñez D, García-Belmonte R, Miorin L, García-Sastre A, Revilla Y (2021) African swine fever virus induces STAT1 and STAT2 degradation to counteract IFN-I signaling. Front Microbiol 12:722952 34512601 10.3389/fmicb.2021.722952 PMC8427279

[19] Zhang KS, Yang B, Shen CC, Zhang T, Hao Y, Zhang DJ, Liu HN, Shi XJ, Li GL, Yang JK, Li D, Zhu ZX, Tian H, Yang F, Ru Y, Cao WJ, Guo JH, He JJ, Zheng HX, Liu XT (2022) MGF360–9L is a major virulence factor associated with the African swine fever virus by antagonizing the JAK/STAT signaling pathway. mBio 13:e0233021 35076286 10.1128/mbio.02330-21 PMC8788333

[20] Huang Z, Cao HX, Zeng FL, Lin SZ, Chen JL, Luo Y, You JY, Kong CY, Mai ZZ, Deng J, Guo WT, Chen XN, Wang H, Zhou P, Zhang GH, Gong L (2023) African swine fever virus MGF505-7R interacts with interferon regulatory factor 9 to evade the type I interferon signaling pathway and promote viral replication. J Virol 97:e0197722 36815839 10.1128/jvi.01977-22 PMC10062159

[21] Ye GQ, Zhang ZX, Liu XH, Liu HY, Chen WY, Feng CY, Li JN, Zhou QQ, Zhao DM, Zhang S, Chen HF, Bu ZG, Huang L, Weng CJ (2024) African swine fever virus pH240R enhances viral replication via inhibition of the type I IFN signaling pathway. J Virol 98:e0183423 38353534 10.1128/jvi.01834-23 PMC10949494

[22] Fan W, Jiao P, Zhang H, Chen T, Zhou X, Qi Y, Sun L, Shang Y, Zhu H, Hu R, Liu W, Li J (2020) Inhibition of African swine fever virus replication by porcine type I and type II interferons. Front Microbiol 11:1203 32655518 10.3389/fmicb.2020.01203 PMC7325991

[23] Paez E, Garcia F, Gil Fernandez C (1990) Interferon cures cells lytically and persistently infected with African swine fever virus in vitro. Arch Virol 112:115–127 1695091 10.1007/BF01348989

[24] Li D, Yang WP, Li LL, Li P, Ma Z, Zhang J, Qi XL, Ren JJ, Ru Y, Niu QL, Liu ZJ, Liu XT, Zheng HX (2021) African swine fever virus MGF-505-7R negatively regulates cGAS-STING-mediated signaling pathway. J Immunol 206:1844–1857 33712518 10.4049/jimmunol.2001110 PMC8023146

[25] Li JN, Song J, Kang L, Huang L, Zhou SJ, Hu L, Zheng J, Li CY, Zhang XF, He XJ, Zhao DM, Bu ZG, Weng CJ (2021) pMGF505-7R determines pathogenicity of African swine fever virus infection by inhibiting IL-1β and type I IFN production. PLoS Pathog 17:e1009733 34310655 10.1371/journal.ppat.1009733 PMC8341718

[26] Ramirez-Medina E, Rai A, Espinoza N, Valladares A, Silva E, Velazquez-Salinas L, Borca MV, Gladue DP (2023) Deletion of the H240R gene in African swine fever virus partially reduces virus virulence in swine. Viruses 15:1477 37515164 10.3390/v15071477 PMC10384018

[27] Li JN, Song J, Zhou SJ, Li S, Liu J, Li TT, Zhang ZX, Zhang XF, He XJ, Chen WY, Zheng J, Zhao DM, Bu ZG, Huang L, Weng CJ (2023) Development of a new effective African swine fever virus vaccine candidate by deletion of the H240R and MGF505-7R genes results in protective immunity against the Eurasia strain. J Virol 97:e0070423 37768081 10.1128/jvi.00704-23 PMC10617561

[28] Vu HLX, McVey DS (2024) Recent progress on gene-deleted live-attenuated African swine fever virus vaccines. NPJ Vaccines 9:60 38480758 10.1038/s41541-024-00845-9 PMC10937926

[29] Cackett G, Matelska D, Sýkora M, Portugal R, Malecki M, Bähler J, Dixon L, Werner F (2020) The African swine fever virus transcriptome. J Virol 94:e0011920 10.1128/JVI.00119-20 PMC7163114 32075923

[30] Chen X, Li LF, Yang ZY, Li M, Fan S, Shi LF, Ren ZY, Cao XJ, Zhang Y, Han S, Wan B, Qiu HJ, Zhang G, He WR (2023) The African swine fever virus I10L protein inhibits the NF-κB signaling pathway by targeting IKKβ. J Virol 97:e0056923 37607059 10.1128/jvi.00569-23 PMC10537781

[31] He WR, Cao LB, Yang YL, Hua D, Hu MM, Shu HB (2021) VRK2 is involved in the innate antiviral response by promoting mitostress-induced mtDNA release. Cell Mol Immunol 18:1186–1196 33785841 10.1038/s41423-021-00673-0 PMC8093274

[32] Sun H, Wu M, Zhang Z, Wang Y, Yang J, Liu Z, Guan G, Luo J, Yin H, Niu Q (2023) OAS1 suppresses African swine fever virus replication by recruiting TRIM21 to degrade viral major capsid protein. J Virol 97:e0121723 37815352 10.1128/jvi.01217-23 PMC10617512

[33] Esparza I, González JC, Viñuela E (1988) Effect of interferon-alpha, interferon-gamma and tumour necrosis factor on African swine fever virus replication in porcine monocytes and macrophages. J Gen Virol 69:2973–2980 3143809 10.1099/0022-1317-69-12-2973

[34] Golding JP, Goatley L, Goodbourn S, Dixon LK, Taylor G, Netherton CL (2016) Sensitivity of African swine fever virus to type I interferon is linked to genes within multigene families 360 and 505. Virology 493:154–161 27043071 10.1016/j.virol.2016.03.019 PMC4863678

[35] Stollar EJ, Smith DP (2020) Uncovering protein structure. Essays Biochem 64:649–680 32975287 10.1042/EBC20190042 PMC7545034

[36] Yu L, Chen Y, Tooze SA (2018) Autophagy pathway: cellular and molecular mechanisms. Autophagy 14:207–215 28933638 10.1080/15548627.2017.1378838 PMC5902171

[37] Dennis MD, Coleman CS, Berg A, Jefferson LS, Kimball SR (2014) REDD1 enhances protein phosphatase 2A-mediated dephosphorylation of Akt to repress mTORC1 signaling. Sci Signal 7:ra68 25056877 10.1126/scisignal.2005103 PMC4145530

[38] Zhong Y, Tian F, Ma HX, Wang HH, Yang W, Liu ZG, Liao AJ (2020) FTY720 induces ferroptosis and autophagy via PP2A/AMPK pathway in multiple myeloma cells. Life Sci 260:118077 32810509 10.1016/j.lfs.2020.118077

[39] Sun JF, Yu HX, Wang YN, Li LM, Zhu JQ, Ma P, Feng ZZ, Tu CC (2023) Classical swine fever virus NS5A protein activates autophagy via the PP2A-DAPK3-Beclin 1 axis. J Virol 97:e0098823 38038430 10.1128/jvi.00988-23 PMC10734420

[40] Zhu RN, Wang Y, Zhang H, Yang JJ, Fan JQ, Zhang YY, Wang Y, Li QX, Zhou XT, Yue HX, Qi Y, Wang SC, Chen T, Zhang SF, Hu RL (2024) Deletion of the B125R gene in the African swine fever virus SY18 strain leads to an A104R frameshift mutation slightly attenuating virulence in domestic pigs. Virus Res 343:199343 38423214 10.1016/j.virusres.2024.199343 PMC10982076

